# Conference Calendar

**DOI:** 10.1002/cfg.439

**Published:** 2004


					Comparative and Functional Genomics
Comp Funct Genom 2004; 5: 552-554.
Published online in Wiley InterScience (www.interscience.wiley.com). DOI: 10.1002/cfg.439
Conference Calendar
February -- April 2005
Animal Models
BSAS -- Annual Meeting, April 4-6
http://www.bsas.org.uk/meetings/annual.htm
Bioinformatics
IASTED -- Databases and Applications,
February 14-16
http://www.iasted.org/conferences/2005/
innsbruck/dba.htm
IBC -- InfoTechPharma, March 15-16
http://www.infotechpharma.com/
KEY -- Proteomics and Bioinformatics, April
8-13
http://www.keystonesymposia.org/Meetings/
ViewMeetings.cfm?MeetingID=734
Functional Genomics
Bologna Winter School: How complex is
functional genomics, February 13-19
http://www.biocomp.unibo.it/school2005/
CHI -- RNAi for Pathway Analysis, March
22-23
http://www.healthtech.com/2005/rpa/index.asp
EuroSciCon -- RNA Mediated Interference in
Practice, April 8
http://www.regonline.co.uk/eventinfo.asp?
EventId=16245
CHI -- Pathway Analysis for Target and
Compound Evaluation, April 20-22
http://www.chimolecularmed.com/05gdd.asp
Human Genomics and Genetics
ICGEB/ESF-FG -- The pathology of pre-mRNA
splicing, April 7-9
http://www.icgeb.org/MEETINGS/CRS05/
ESF.htm
HUGO -- Human Genome Meeting
(HGM2005), April 18-21
http://hgm2005.hgu.mrc.ac.uk/
General
ABRF -- Biomolecular Technologies:
Discovery to Hypothesis, February 5-8
http://www.faseb.org/meetings/abrf2005/
default.htm
CASSS -- MicroScale Bioseparations, February
12-17
http://www.casss.org/meetings/2005msb/
default.htm
GRC -- Quantitative Genetics and Genomics,
February 20-25
http://www.grc.uri.edu/programs/2005/
quantgen.htm
Experimental Biology, April 2-6
http://www.faseb.org/meetings/eb2005/call/
default.htm
ASBMB-2005 Annual Meeting, April 2-6
http://asbmb.interliant.com/asbmb/site.nsf/Sub/
2005AnnualMeeting
Microbes
SGM-156th Meeting, April 4-7
http://www.sgm.ac.uk/meetings/MTGPAGES/
hw.cfm
Copyright  2004 John Wiley & Sons, Ltd.
Conference Calendar 553
TIGR -- Genomes 2005: April 13-16
http://www.tigr.org/conf/
ASM -- Beneficial Microbes, April 17-21
http://www.asm.org/Meetings/index.asp?
bid=27608
Pharmacogenomics/Genomics and
Disease
AACR -- Oncogenomics 2005: Dissecting
Cancer through Genome Research, February
2-6
http://www.aacr.org/2005oncogenomics/
index.html
CHI -- Biomarker Validation and Translational
Research, February 7-9
http://www.healthtech.com/2005/bmv/index.asp
IBC -- Clinical Genomics, February 17-18
http://www.ibclifesciences.com/3103/?source=
3103-17
IBC -- Biomarker Pipeline: From Discovery to
Clinical Trials, March 14-16
http://www.ibclifesciences.com/biomarkers
IBC -- Drug Discovery Technology Europe,
March 14-17
http://www.drugdisc.com/europe/
CHI -- Glycomics and Carbohydrates in Drug
Development, March 21-22
http://www.healthtech.com/2005/gly/index.asp
CHI -- Molecular Diagnostics, April 20-22
http://www.chimolecularmed.com/05mdx.asp
Plants
GRC -- Temperature Stress in Plants, January
30-February 4
http://www.grc.uri.edu/programs/2005/
tempstrs.htm
KEY -- Plant Cell Signaling: In vivo and Omics
Approaches, February 1-6
http://www.keystonesymposia.org/Meetings/
ViewMeetings.cfm?MeetingID=727
47th Maize Genetics Conference, March 10-13
http://www.maizegdb.org/maize meeting/2005/
SEB -- Plant Frontier Meeting, March 21-23
http://www.sebiology.org/Animal/pageview.asp?
S=2&mid=83
Proteomics
KEY -- Proteomics and Bioinformatics, April
8-13
http://www.keystonesymposia.org/Meetings/
ViewMeetings.cfm?MeetingID=734
VIth European Symposium of The Protein
Society, April 30-May 4
http://www.proteinsociety2005.info/barcelona.
html
Structural Genomics
KEY -- Frontiers of NMR in Molecular Biology
IX, January 29-February 4
http://www.keystonesymposia.org/Meetings/
ViewMeetings.cfm?MeetingID=752
Systems Biology
CSHL -- Systems Biology: Global Regulation of
Gene Expression, March 17-20
http://meet1.cshl.edu/meetings/systems05.shtml
KEY -- Systems and Biology, April 8-13
http://www.keystonesymposia.org/Meetings/
ViewMeetings.cfm?MeetingID=733
Copyright  2004 John Wiley & Sons, Ltd. Comp Funct Genom 2004; 5: 552-554.
554 Conference Calendar
Transcriptomics/Regulation
CHI -- Quantitative PCR, March 21-22
http://www.healthtech.com/2005/qpc/index.asp
KEY -- Chromatin Modification Pathways,
March 31-April 5
http://www.keystonesymposia.org/Meetings/
ViewMeetings.cfm?MeetingID=745
BS-Translation UK, April 13-15
http://www.biochemistry.org/meetings/
programme.cfm?Meeting No=SA035
Biotech Industry
Biomedex 2005: Canadian Biotechnology
Conference, March 30-31
http://www.biomedex.info/
IBC -- European BioProcess International,
April 12-13
http://www.ibc-lifesci.com/bpi/
ACS/BIO/NABC -- World Congress on
Industrial Biotechnology and Bioprocessing,
April 20-22
http://www.bio.org/worldcongress/
Key:
AACR: American Association for Cancer Re-
search: http://www.aacr.org/
ABRF: Association of Biomolecular Resource
Facilities: http://www.abrf.org/
ACS: American Chemical Society: http://www.
acs.org/
ASBMB: American Society for Biochemistry and
Molecular Biology: http://www.asbmb.org/
ASM: American Society for Microbiology:
http://www.asm.org/
BIO: Biotechnology Industry Organization:
http://www.bio.org/
BS: Biochemical Society: http://www.
biochemistry.org/
BSAS: British Society of Animal Science:
http://www.bsas.org.uk
CASS: California Separation Science Society:
http://www.casss.org/
CHI: Cambridge Healthtech Institute: http:
//www.healthtech.com
CSHL: Cold Spring Harbor Laboratories:
http://meetings.cshl.org/index.htm
ESF-FG: European Science Foundation Programme
in Functional Genomics:
http://www.functionalgenomics.org.uk/
EuroSciCon: http://www.euroscicon.com/
GRC: Gordon Research Conferences:
http://www.grc.uri.edu/
HUGO: Human Genome Organisation:
http://www.gene.ucl.ac.uk/hugo/
IASTED: International Association of Science and
Technology for Development: http://www.
iasted.org/
IBC: IBC Conferences: http://www.ibc-lifesci.com
ICGEB: International Centre for Genetic Engineer-
ing and Biotechnology: http://www.icgeb.
org/index.htm
KEY: Keystone Symposia: http://www.
keystonesymposia.org/
NABC: National Agricultural Biotechnology
Council: http://nabc.cals.cornell.edu/
SEB: Society for Experimental Biology:
http://www.sebiology.org/
SGM: Society for General Microbiology:
http://www.sgm.ac.uk/
TIGR: The Institute for Genomic Research:
http://www.tigr.org/
The Conference Calendar is a listing of forthcoming conferences of interest to our readership. We provide
the title, dates and web address of each conference, to enable readers to find out more information.
Inclusion does not imply endorsement by the journal. If you know of a relevant conference, please
contact the Managing Editor.
Copyright  2004 John Wiley & Sons, Ltd. Comp Funct Genom 2004; 5: 552-554.

				

